# Functional Analysis of Durum Wheat GASA1 Protein as a Biotechnological Alternative Against Plant Fungal Pathogens and a Positive Regulator of Biotic Stress Defense

**DOI:** 10.3390/plants14010112

**Published:** 2025-01-02

**Authors:** Mohamed Taieb Bouteraa, Walid Ben Romdhane, Alina Wiszniewska, Narjes Baazaoui, Mohammad Y. Alfaifi, Anis Ben Hsouna, Miroslava Kačániová, Stefania Garzoli, Rania Ben Saad

**Affiliations:** 1 Biotechnology and Plant Improvement Laboratory, Centre of Biotechnology of Sfax, University of Sfax, P.O. Box 1177, Sfax 3018, Tunisia; bouteraa.taieb@gmail.com (M.T.B.); benhsounanis@gmail.com (A.B.H.); raniabensaad@gmail.com (R.B.S.); 2 Faculty of Sciences of Bizerte UR13ES47, University of Carthage, Zarzouna, Bizerte 7021, Tunisia; 3 Plant Production Department, College of Food and Agricultural Sciences, King Saud University, P.O. Box 2460, Riyadh 11451, Saudi Arabia; walid.brm3@gmail.com; 4 Department of Botany, Physiology and Plant Protection, University of Agriculture in Kraków, Al. Mickiewicza 21, 31-120 Kraków, Poland; alina.wiszniewska@urk.edu.pl; 5 Biology Department, Faculty of Science, King Khalid University, Abha 61421, Saudi Arabia; nhrmbaazoui@kku.edu.sa (N.B.); alfaifi@kku.edu.sa (M.Y.A.); 6 Department of Environmental Sciences and Nutrition, Higher Institute of Applied Sciences and Technology of Mahdia, University of Monastir, Mahdia 5100, Tunisia; 7 Institute of Horticulture, Faculty of Horticulture and Landscape Engineering, Slovak University of Agriculture, Tr. A. Hlinku 2, 94976 Nitra, Slovakia; miroslava.kacaniova@gmail.com; 8 School of Medical and Health Sciences, University of Economics and Human Sciences in Warsaw, Okopowa 59, 01-043 Warszawa, Poland; 9 Department of Chemistry and Technologies of Drug, Sapienza University, P.le Aldo Moro 5, 00185 Rome, Italy

**Keywords:** stress tolerance, defense signaling, TdGASA1 protein, *Triticum durum*, antifungal activity, transgenic *Arabidopsis*

## Abstract

Plants are frequently challenged by a variety of microorganisms. To protect themselves against harmful invaders, they have evolved highly effective defense mechanisms, including the synthesis of numerous types of antimicrobial peptides (AMPs). Snakins are such compounds, encoded by the *GASA* (Gibberellic Acid-Stimulated Arabidopsis) gene family, and are involved in the response to biotic and abiotic stress. Here, we examined the function of the newly identified TdGASA1 gene and its encoded protein in *Triticum durum* subjected to different biotic stress-related simulants, such as mechanical injury, methyl jasmonate (MeJA), indole-3-acetic acid (IAA), salicylic acid (SA), hydrogen peroxide (H_2_O_2_), as well as infection with pathogenic fungi *Fusarium graminearum* and *Aspergillus niger*. We found that in durum wheat, *TdGASA1* transcripts were markedly increased in response to these stress simulants. Isolated and purified TdGASA1 protein exhibited significant antifungal activity in the growth inhibition test conducted on eight species of pathogenic fungi on solid and liquid media. Transgenic *Arabidopsis* lines overexpressing *TdGASA1* obtained in this study showed higher tolerance to detrimental effects of H_2_O_2_, MeJA, and ABA treatment. In addition, these lines exhibited resistance to *Fusarium graminearum* and *Aspergillus niger*, which was linked to a marked increase in antioxidant activity in the leaves under stress conditions. This resistance was correlated with the upregulation of pathogenesis-related genes (*AtPDF1.2a*, *AtERF1*, *AtVSP2*, *AtMYC2*, *AtPR1*, *AtACS6*, *AtETR1*, and *AtLOX2*) in the transgenic lines. Overall, our results indicate that TdGASA1 gene and its encoded protein respond ubiquitously to a range of biotic stimuli and seem to be crucial for the basal resistance of plants against pathogenic fungi. This gene could therefore be a valuable target for genetic engineering to enhance wheat resistance to biotic stress.

## 1. Introduction

Antimicrobial peptides (AMPs) are crucial components of the immune system across a wide range of living organisms, including microorganisms, arthropods, plants, and animals [1]. These protein-based compounds are essential for protection against a variety of pathogens, such as Gram-negative and Gram-positive bacteria, viruses, fungi, and other parasites [2,3]. In plants, AMPs are key elements of innate immunity, functioning as essential peptides in plant defense mechanisms. Many AMPs are rich in cysteine and are integral to defense systems against infections [4]. Within plants, several AMPs have been identified, including snakins (SN)/GASA (Gibberellic Acid-Stimulated Arabidopsis) proteins, thionins, defensins, knottins, cyclotides, puroindolines, lipid transfer proteins, and hevein-type proteins [4,5]. The *Snakin-1* and *Snakin-2* genes were first identified in potatoes and were shown to possess antimicrobial properties [6,7]. According to Almasia et al. [8], overexpression of the *Snakin-1* gene in potatoes resulted in enhanced resistance to *Rhizoctonia solani* and *Erwinia carotovora* in vitro.

Another study indicated that the *Snakin-1* gene plays a crucial role in maintaining redox balance (ROS), regulating ascorbic acid levels, and facilitating hormonal crosstalk in potatoes [9]. In transgenic lettuce plants (*Lactuca sativa* L.), overexpressing the potato *Snakin-1* gene enhanced their resistance to *Rhizoctonia solani* and *Sclerotinia sclerotiorum* [10]. Furthermore, the knockdown of the potato *Snakin-1* (*StSN1*) gene was shown to impact cell wall composition, cell division, and primary metabolism in leaves, indicating that *StSN1* may be involved in a variety of cellular processes [11]. GASA proteins are intriguing biotechnological targets due to their valuable biological properties, making them particularly promising for developing new disease control agents [12]. Heterologous expression is a commonly used technique for medium- and large-scale production of proteins, allowing for the efficient and cost-effective production of various proteins in hosts such as bacteria, yeast, fungi, and plants. Several production systems have been developed for this purpose [13,14,15].

Notably, recombinant proteins have been successfully produced in *Escherichia coli*, *Pichia pastoris*, and more recently, in baculovirus/insect cells, retaining their expected antibacterial and antifungal properties [16,17,18,19]. Moreover, a synthetic *Snakin-1*, derived from the potato *StSN1*, has recently shown a marked inhibitory effect against specific food spoilage yeasts without any risk to human consumption [19]. In our previous study, we identified 19 genes belonging to the *GASA* family in durum wheat (*Triticum durum*) and found that one of these genes, termed *TdGASA1*, was notably upregulated in response to salinity, osmotic stress, and treatment with exogenous phytohormones, specifically ABA and GA3 [20].

We also observed that the heterologous expression of the *TdGASA1* gene in *Saccharomyces cerevisiae* enhanced the yeast’s tolerance to a range of stress factors. On the basis of these results, we hypothesized that this gene and its encoded protein may play a role in mechanisms that confer ubiquitous tolerance to various stresses. Therefore, in this study, we investigated the expression profile of the *TdGASA1* gene in response to biotic stress-related stimuli and pathogen infection in durum wheat. Moreover, the isolated and purified TdGASA1 protein was examined for its antifungal activity with a view to elaborate novel sustainable solutions for plant disease management and crop protection. The detailed workflow of the present study is presented in Supplementary Figure S1.

We also aimed at developing transformation system for analyzing the expression of the *TdGASA1* in transgenic *Arabidopsis thaliana*, to provide mechanistic insight into the role and the function of this representative gene belonging to GASA family in modulating the responses to biotic stress in higher plants.

## 2. Results

### 2.1. TdGASA1 Gene in Durum Wheat Is Highly Induced by Multiple Biotic Stress Simulators

**Figure 1:**
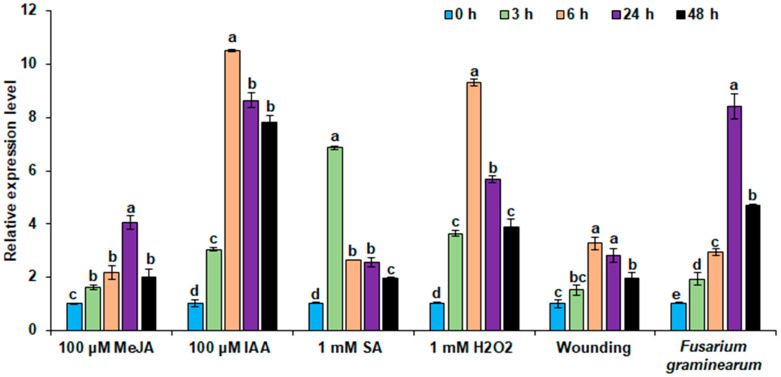
qRT-PCR analysis of *TdGASA1* transcript levels in durum wheat seedlings exposed to various biotic stress simulators. One-week-old wheat seedlings were subjected to wounding and treated with 100 μM MeJA, 100 µM IAA, 1 mM SA, 1 mM H_2_O_2_ and after inoculation with *Fusarium graminearum* for 3, 6, 24 and 48 h. The *CDC* gene was used as an internal control. Vertical bars represent the standard deviation calculated from three replicates. The values are expressed as mean ± SD (*n* = 3). Means that share the same letter do not differ significantly at *p* < 0.05.

To investigate the involvement of *TdGASA1* gene in host defense responses, we examined its expression pattern during the first two days after the application of biotic stress-related stimuli: signaling molecules MeJA, IAA, SA, and H_2_O_2_, mechanical wounding and after infection with *Fusarium graminearum*. Transcript levels were analyzed in the leaves of durum wheat Karim at 3, 6, 24, and 48 h. As presented in Figure 1, *TdGASA1* expression was induced under all stress treatments, but the accumulation of transcripts differed significantly among the applied stimulants. In the case of MeJa, the highest accumulation of *TdGASA1* transcripts was observed after 24 h, reaching four times the level of the control, and declined at 48 h (Figure 1).

IAA and H_2_O_2_ application induced the highest expression after 6 h, amounting to 10-fold. However, at subsequent time points, the transcript level remained rather high in the case of IAA, and significantly dropped in the case of H_2_O_2_ (Figure 1). The highest *TdGASA1* expression after SA application was determined at 3 h, but then the transcript level quickly declined and remained stable until 48 h (Figure 1). Wounding induced TdGASA1 expression much less than signaling molecules, and the highest transcript accumulation was determined at 6 and 24 h. It means that the maximal level was maintained at least for 18 h, which was not observed in any other tested treatment. Considering the effect of fungal infection, *TdGASA1* expression was the highest one-day post-inoculation, when transcript level increased eight-fold (Figure 1). Subsequently, it decreased to approximately four-fold but remained significantly higher than in the mock control.

### 2.2. Overexpression and Purification of Recombinant TdGASA1 Protein

**Figure 2:**
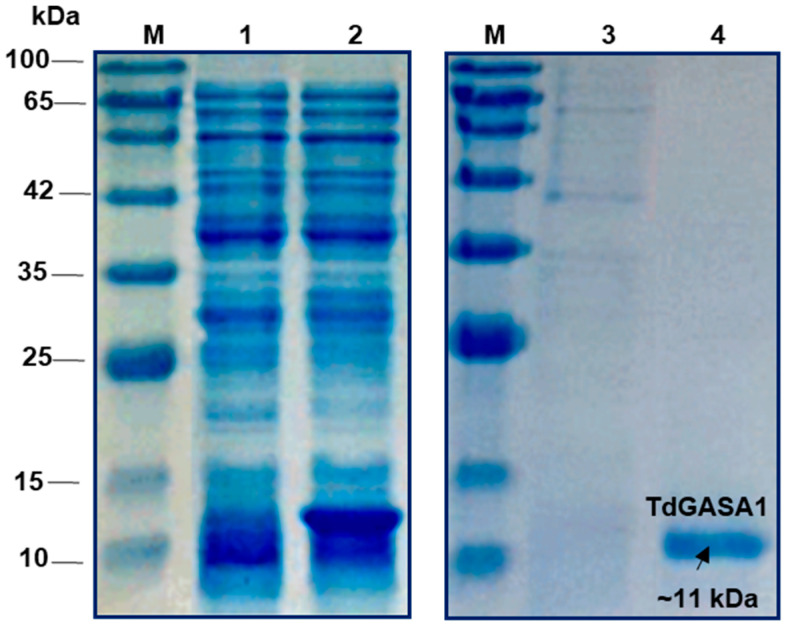
SDS-PAGE of TdGASA1 recombinant protein. The TdGASA1 protein was expressed in *E. coli* and subsequently purified. The soluble and purified fractions from TdGASA1-expressing *E. coli* BL21 cells were analyzed using 12% SDS-PAGE, with the gel stained using Coomassie Blue. Lane M: Low molecular weight marker, Lane 1: pET28a-TdGASA1 without IPTG induction, Lane 2: pET28a-TdGASA1 with IPTG induction, Lane 3: flow through, and Lane 4: purified recombinant TdGASA1 proteins.

The *TdGASA1* nucleotide sequence was amplified using gene-specific primers. Its open reading frame (ORF) was then inserted into the pET28a (+) expression vector. Following induction with IPTG, the recombinant TdGASA1 fusion protein was produced in large amounts in *E. coli* BL21 cells (Figure 2). SDS-PAGE analysis confirmed that the molecular weight of the recombinant TdGASA1 was approximately 11 kDa when compared to non-induced cells. The recombinant TdGASA1 protein underwent purification, during which the His tag was cleaved and removed. The purified recombinant TdGASA1 yield was 0.55 mg/mL.

### 2.3. Antifungal Activity

The results showed that TdGASA1 protein exerted significant and strong inhibitory effect on the growth of *Fusarium graminearum* (ISPAVE 271) and *Aspergillus flavus* (food isolate), with diameter of inhibition zone amounting to 20 and 22 mm and MIC values of 312 µg/mL, respectively (Table 1). Also, the TdGASA1 protein exhibited an antifungal activity against *Fusarium oxysporum* (CTM10402) and *Aspergillus* sp., including *Aspergillus niger* (CTM 10099) and *Aspergillus flavus* (food isolate).

The maximal inhibition zone diameters were 18–22 mm, and MIC values ranged from 0.078 to 1.25 mg/mL (Table 1). The inhibition zone of the purified protein against *Aspergillus* sp. was measured as 15–22 mm and the minimal inhibition concentration values amounted to 0.312–1.25 mg/mL.

### 2.4. Production of Transgenic Arabidopsis Plants Overexpressing TdGASA1

**Figure 3:**
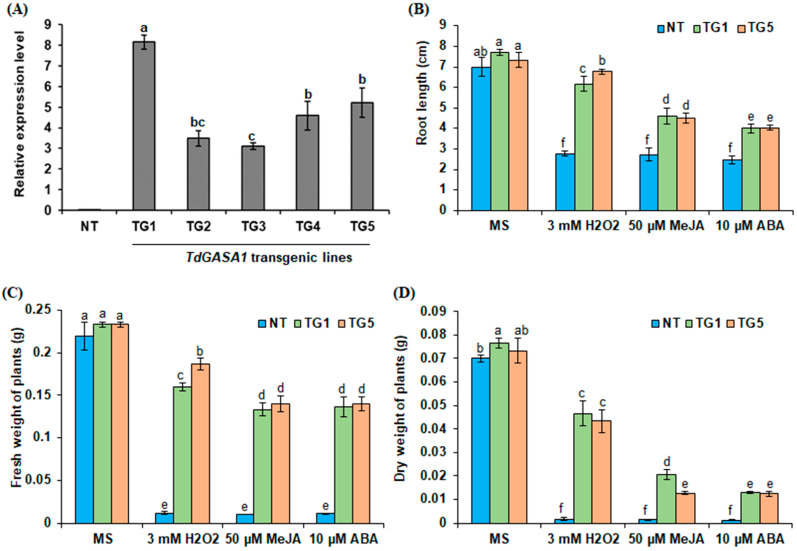
Effect of stress treatments on growth of NT and transgenic *Arabidopsis* plants. (**A**) Relative quantification of *TdGASA1* transcript accumulation in both engineered and NT plants. (**B**) Root length, (**C**) fresh weight, and (**D**) dry weight of seedlings grown on control medium (MS/2) or MS/2 medium supplemented with H_2_O_2_ (3 mM), MeJA (50 µM), or ABA (10 µM) for 15 days. Results are presented as mean ± SEM from three technical replicates. Means that share the same letter are not significantly different at *p* < 0.05.

In the transformation experiment, several transgenic lines of *Arabidopsis thaliana* were generated and advanced to the T3 generation, from which homozygous plants were selected. RT-qPCR analysis showed that the accumulation of *TdGASA1* transcripts was higher in TG1 and TG5 lines than in the other lines (TG2, TG3 and TG4) (Figure 3A). Therefore, for further investigation of the physiological role of *TdGASA1* gene, TG1 and TG5 transgenic lines, exhibiting higher expression levels of the transgene, were chosen.

### 2.5. The Overexpression of TdGASA1 Enhances Tolerance to H_2_O_2_, ABA and MeJA Treatments

**Figure 4:**
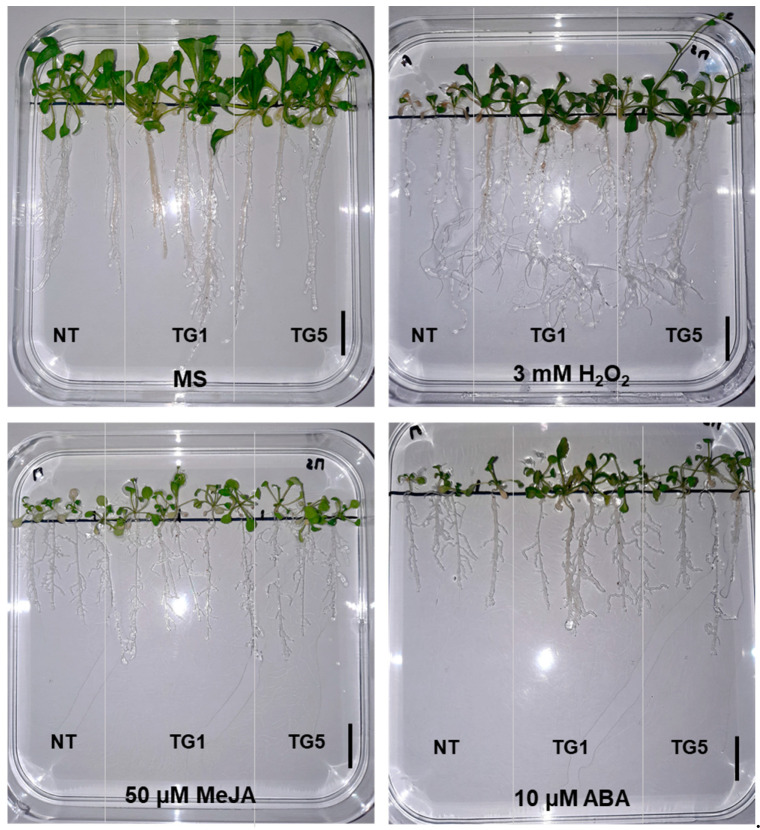
Phenotype of seedlings following six days of treatment with oxidative stress (3 mM H_2_O_2_) and hormonal treatments (50 µM MeJA or 10 µM ABA) during germination. Scale bars represent 1 cm.

The aim of this experiment was to evaluate the effects exerted by signaling molecules on growth performance in the lines overexpressing *TdGASA1* (Figure 3 and Figure 4). Under standard growth conditions, the two transgenic *Arabidopsis* lines (TG1 and TG5) displayed a phenotypic appearance similar to that of NT plants (Figure 4). However, under external stimuli, the transgenic plants overexpressing TdGASA1 displayed greater tolerance on the morphological level than NT plants (Figure 4). In fact, the *TdGASA1* transgenic lines continued to grow well in the presence of 3 mM H_2_O_2_ whereas NT plants developed chlorosis and died within 15 days of stress treatment. A difference between transgenic and NT plants subjected to 10 µM ABA was also observed (Figure 4). The transgenic lines continued to grow without disturbance, whereas NT plants became chlorotic (Figure 4). When exposed to MeJA, NT plants grew slower, while the transgenic lines continued to grow normally (Figure 4). The changes in growth performance under respective compounds was confirmed by measuring the root length (Figure 3B), shoot fresh weight (Figure 3C) and dry weight (Figure 3D). In the absence of stress, both transgenic and NT plants had comparable root lengths, fresh weights of shoots, and dry weights. However, upon exposure to various treatments, the NT plants exhibited a significant reduction in all biometrical parameters studied (Figure 3B–D). Both transgenic lines responded similarly to each other and experienced less pronounced reductions in these parameters. Of note, MeJa and ABA had more negative impact on growth performance than H_2_O_2_ treatment (Figure 3B–D).

### 2.6. TdGASA1-Arabidopsis Plants Confer Resistance to Fungal Infection by the Induction of Antioxidant Response and Modulation of Pathogenesis-Related Genes Expression

**Figure 5:**
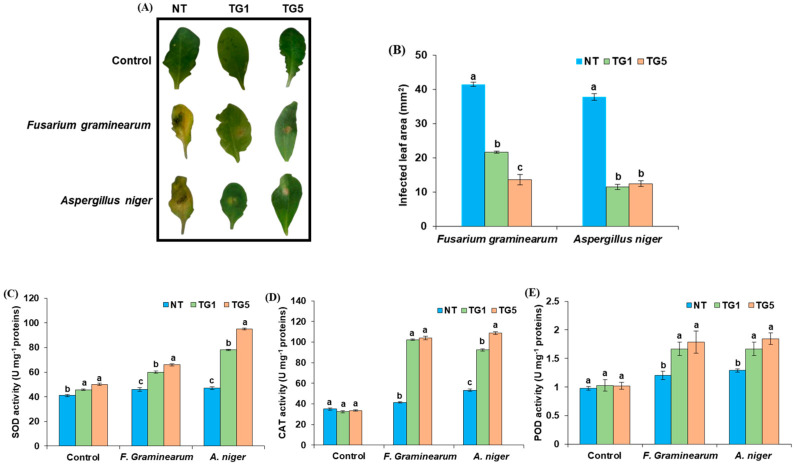
Resistance of transgenic *Arabidopsis* leaves overexpressing the *TdGASA1* gene to biotic stress. (**A**) Detached leaves from both NT and *TdGASA1* transgenic *Arabidopsis* plants were observed six days after inoculation (dpi). (**B**) The area of infection on the detached leaves from NT and transgenic *Arabidopsis* was quantified. Additionally, the impact of pathogen infection on the activity of antioxidant enzymes, including (**C**) superoxide dismutase (SOD), (**D**) catalase (CAT), and (**E**) peroxidase (POD), was assessed in the leaves of both transgenic and NT *Arabidopsis thaliana* plants. The results represent the means from three replicates, with each replicate taken from one fully expanded leaf per plant. Statistically significant differences are indicated by different letters (*p* < 0.05).

Given that TdGASA1 exhibits antifungal activity in vitro, we aimed to explore the resistance to fungal infections in two lines of transgenic *Arabidopsis* expressing *TdGASA1* (TG1 and TG5), conducting detached leaf assays with phytopathogenic fungi *Fusarium graminearum* and *Aspergillus niger*. On the leaves of NT plants, spreading lesions with an area of 35–40 mm^2^ developed at the site of contact (Figure 5A). In contrast, the leaves from TdGASA1-expressing transgenic plants had smaller (10–20 mm^2^), more localized lesions around the contact site (Figure 5A). These observations suggested that the presence of TdGASA1 reduced proliferation/expansion of *Fusarium* and *Aspergillus* cells and significantly restricted their spreading within leaf tissue. In infected leaves of NT and transgenic lines, we compared the activities of antioxidant enzymes: catalase (CAT), superoxide dismutase (SOD), and peroxidase (POD). The results show that following the infection, the activities of all the enzymes in transgenic plants were significantly boosted in comparison with NT plants, with CAT being the most inducible by pathogen infection (Figure 5B–E). Reactions of both transgenic lines to both pathogenic fungi were similar, indicating that observed modulation of antioxidant activity could be of ubiquitous character related to the expression of *TdGASA1*.

**Figure 6:**
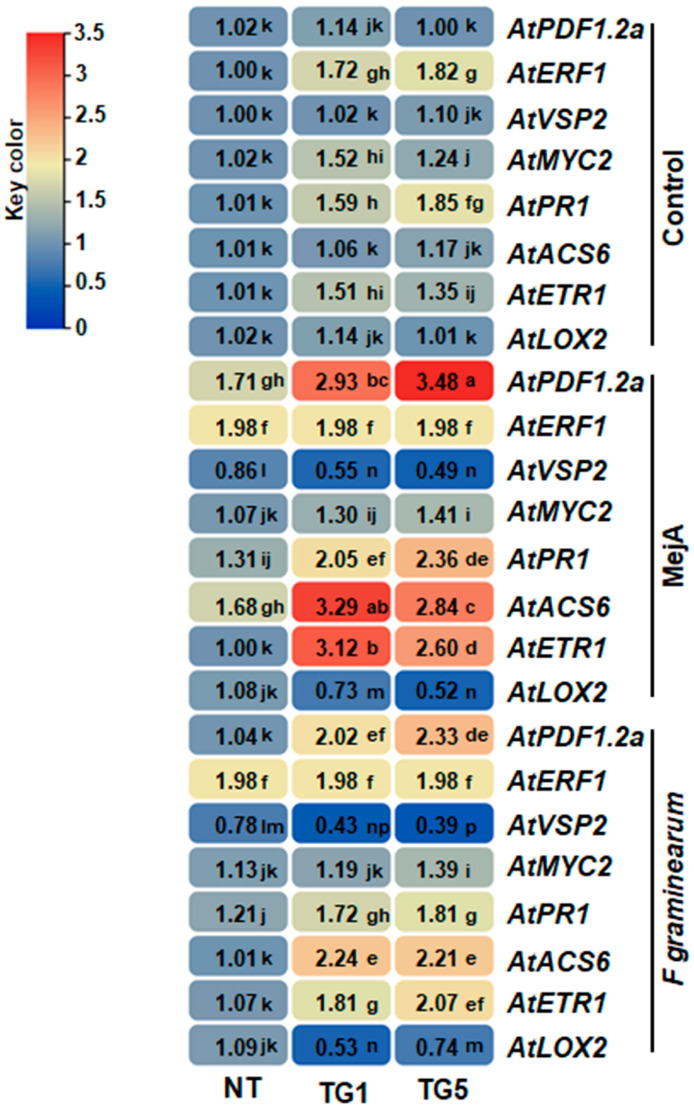
A heatmap illustrating the expression profiles of defense-related genes (*AtPDF1.2a*, *AtERF1*, *AtVSP2*, *AtMYC2*, *AtPR1*, *AtACS6*, *AtETR1*, and *AtLOX2*) was generated for transgenic *Arabidopsis* lines (TG1 and TG5) and NT plants, both before and after infection with *F. graminearum* for 3 days and treatment with 50 µM MeJA for 15 days. Different letters indicate that the corresponding mean values are different (*p* < 0.05) according to Duncan test.

To further explore the molecular mechanisms by which TdGASA1 influenced plant response to biotic stress, we compared the expression profiles of eight biotic stress-related genes (*AtPDF1.2a*, *AtERF1*, *AtVSP2*, *AtMYC2*, *AtPR1*, *AtACS6*, *AtETR1*, and *AtLOX2*) between NT and *TdGASA1*-transgenic lines. As illustrated in Figure 5, the expression levels of *PR1*, *MYC2*, *ACS6*, *ETR1*, and *PDF1.2a* were significantly higher in the transgenic lines TG1 and TG5 following MeJA treatment and *Fusarium* infection. Conversely, the expression of *VSP2* and *LOX3* genes was reduced in the two *TdGASA1* transgenic lines compared to NT plants (Figure 6).

## 3. Discussion

The Gibberellic Acid-Stimulated Arabidopsis (*GASA*) gene family has garnered attention due to its involvement in plant growth, development, and defense mechanisms [21]. The role of *GASA* genes in pathogen response is of particular interest, as emerging research highlights their involvement in modulating plant immune responses and enhancing resistance to pathogens [6,7,8]. In addition to their regulatory functions, GASA proteins may also play a structural role in plant defense. The conserved cysteine-rich domains found in GASA proteins are similar to those in antimicrobial peptides (AMPs), which are known to directly inhibit pathogen growth by disrupting cell membranes or interfering with pathogen metabolism [22,23]. The presence of these domains suggests that GASA proteins might have a similar role in directly combating pathogens [24]. Although the function of the *GASA* gene has been well explored in several plant species [23], the primary mechanism of *TdGASA1* gene action and its involvement in plant defense against biotic stress remain unclear. This study focuses on investigating the potential role of the TdGASA1 gene from durum wheat in stress tolerance. Our findings indicate that *TdGASA1* is regulated by biotic stress signals and plays a significant role in modulating signaling pathways involved in the defense response, thereby enhancing basal resistance. This newly discovered function of *TdGASA1* adds to our understanding of how plants respond to various environmental stresses. In our study, the expression of *TdGASA1* increased in response to biotic stress inducers, including wounding, infection by *F. graminearum*, and signaling molecules like MeJA, SA, IAA and H_2_O_2_. One of the critical ways *GASA* genes mediate plant defense is through their interaction with hormone signaling pathways, particularly gibberellic acid (GA), abscisic acid (ABA), and salicylic acid (SA) [11,25,26]. These hormones play vital roles in regulating plant immune responses. However, previous study indicates that certain *GASA* genes, such as *GASA5*, can interact with both GA and ABA signaling pathways, balancing growth and defense mechanisms in plants [27]. For instance, in *Hevea brasiliensis*, *HbGASA* genes were found to be upregulated in response to fungal infections, suggesting their role in enhancing disease resistance [28]. While a previous study reported that *TdGASA1* transcript levels are relatively unresponsive to GA and ABA [20], our findings suggest a different outcome for other phytohormones. OsGSR1, a member of the rice *GASA* gene family and a positive regulator of GA signaling, has been found to interact with DIM/DWF1, thereby influencing brassinosteroid (BR) synthesis. It also plays a key role in modulating the crosstalk between BR and GA signaling pathways [29]. Both MeJA and auxin appear to induce upregulation of *TdGASA1*, with transcript levels peaking at 24 h following MeJA treatment and at 6 h after IAA exposure. This result is consistent with studies on three *GASA* genes from *Hevea brasiliensis* (*HbGASA7-1*, *HbGASA14-3*, and *HbGASA15*), which are primarily induced by jasmonic acid, auxin, and salicylic acid [28]. These phytohormones are essential for plant defense against biotic stress, especially in response to fungal infections [28]. These findings indicate that *TdGASA1* genes may participate in the regulation of immune responses, potentially by modulating hormone signaling pathways or strengthening cell walls to inhibit pathogen invasion. The upregulation of *GASA* genes in response to wounding has been noted since their initial identification [7,30]. GASA proteins appear to play a crucial role in repair mechanisms following injury, as demonstrated by the functions of *GIP2* and *AtGASA4*, which help reduce the accumulation of hydrogen peroxide (H_2_O_2_) and nitric oxide (NO) in damaged leaves [31,32]. Similarly, in *Gerbera hybrida*, the expression of *PRGL*, a member of the *GASA* gene family, was significantly elevated after mechanical wounding [33].

Recent research highlights the significance of interactions between plant-derived antimicrobial proteins and microbial membranes as a primary mechanism of action [34,35]. These proteins, which typically possess a positive charge, interact with negatively charged bacterial membranes, leading to increased permeability and rapid cell death [36]. Their efficacy is influenced by several factors, including sequence, size, charge, hydrophobicity, and binding affinity [37,38]. The negatively charged microbial surfaces and the phospholipid head groups of the double membrane facilitate the penetration of these peptides [39,40]. Our research indicates the role of positively charged peptides in disrupting bacterial membrane integrity, although the exact bactericidal mechanisms remain unclear and require further investigation. Numerous studies have examined the antibacterial properties of plant-derived antimicrobial peptides and proteins, including antimicrobial plant peptides (PAMPs) and cysteine-rich peptides. These compounds demonstrate broad-spectrum activity against bacteria, fungi, and viruses, indicating significant potential for drug development and applications in the food industry [41,42]. A particular challenge for pharmaceutical and food production is contamination with *Aspergillus*, *Fusarium* and *Alternaria* species, and their respective mycotoxins. In this study, we specifically conducted tests on a range of fungal species being important pathogens to various crop plants. By testing the TdGASA1 against a wide range of pathogenic fungi, we were capable of demonstrating the protein’s broad-spectrum activity and its potential applicability in agricultural practice. Moreover, the results obtained in this study show the potential of GASA proteins in developing new therapeutics and the purposefulness of further exploration in this field [34,43].

We overexpressed *TdGASA1* in *Arabidopsis* to find out whether its role in biotic stress tolerance is ubiquitous. As it was known from previous report on wheat, *TaGASR1* gene, belonging to GASA family, contributed to elevated tolerance to heat and oxidative stress [44]. Our findings showed that *TdGASA1* overexpression modulated the reaction to both oxidative and hormonal stimuli, allowing for undisturbed or ameliorated growth of transgenic seedlings. Additionally, those lines exhibited improved resistance to fungal pathogens *F. gramenarium* and *A. niger*. Our results are in line with numerous previously reported findings demonstrating enhanced resistance of potato, lettuce, tomato, and *Peltophorum dubium* to a wide range of both fungal and bacterial infections [8,10,45]. The responses observed in our study were associated with the induction of antioxidant activity (as revealed by examination of CAT, POD and SOD activity), and changes in expression of several pathogenesis-related genes. Interestingly, GASA proteins themselves may exert antioxidant properties, taking part in H_2_O_2_ elimination, as was discovered in willow [46]. This could be attributed to cysteine-rich domain in their structure, which allows for the involvement of GASA proteins in redox reactions [31]. The decrease in ROS production was also confirmed in *Arabidopsis* lines overexpressing GASA14 gene [47].

Considering genetic regulation, *GASA* genes could enhance plant disease resistance by the activation of defense-related genes [23]. Here we found that *TdGASA1* upregulated the expression of JA/ET-responsive genes (*AtPdF1.2a*, *AtMYC2*, and *AtERF1*), SA-responsive genes (*AtPR-1*), ET-biosynthesis genes (*AtACS6*), and ET-signaling genes (*AtETR1*) in transgenic *Arabidopsis* under pathogen infection with *F. graminearum* and MeJA treatment. Given that it is widely recognized that that salicylic acid (SA) and jasmonic acid (JA) are key signaling molecules in plant defense against pathogens and insects, it is possible that *TdGASA1* influences these defense responses in *Arabidopsis* through the downregulation of certain JAZ-encoding genes (such as *LOX3* and *VSP2*). Previous research has shown that *MYC2* regulates two distinct JA signaling pathways antagonistically, controlling responses to either pathogens or wounding [48]. Thus, *TdGASA1* may have impacted the function of *MYC2*, leading to an increased induction of the *ERF1* and *PDF1.2* genes in response to jasmonic acid (JA), while compromising the expression of the *VSP2* and *LOX3* genes in the transgenic lines. Similarly, overexpression of *GASA4* in *Arabidopsis thaliana* was found to enhance resistance to *Pseudomonas syringae*, a bacterial pathogen. This increased resistance was linked to the upregulation of defense genes such as *PR1* and *PDF1.2* implicated in plant immune responses [49].

## 4. Materials and Methods

### 4.1. Plant Materials and Stress Treatments

Durum wheat seeds (cv. Karim) were obtained from the “Centre d’Appui Chebika-CRDA Kairouan” in Tunisia. Seed sterilization and germination procedures for durum wheat were described by Ben Romdhane et al. [50] and grown in a Phytotron at 25 ± 5 °C, under light intensity of 280 µmol m^−2^ s^−1^, a 16 h photoperiod and 60 ± 10% relative humidity. Following one week of growth in a nutrient solution as detailed by Ben Romdhane et al. [50], the leaves of plants were sprayed with various chemical treatments: (100 µM MeJA, 100 µM IAA, and 1 mM SA) and with water used as the control. The concentrations of phytohormones and H_2_O_2_ used in the present study were chosen based on Ben Saad et al. [51], except for IAA where the chosen concentration was based on Qu et al. [49].The middle section of the leaf was wounded by making an incision with a scalpel. A solution of H_2_O_2_ at a concentration of 1 mM was applied by immersing the roots of the seedlings in the H_2_O_2_ solution. Fully expanded leaves and roots were collected at 3, 6, 24, and 48 h post-treatment for molecular analyses. For the infection experiment, the *F. graminearum* strain [52] was cultivated on potato dextrose agar (PDA) plates for seven days at 30 °C. Fifteen-day-old plants were then treated with a freshly prepared spore suspension (2 × 10^7^ spores/mL) in Hoagland’s solution. After inoculation, all plants were maintained under high humidity conditions at 25 °C with a 16 h photoperiod. Leaf samples were harvested at various time points (3, 6, 24 and 48 h) for each treatment. Fully developed leaves were collected, immediately frozen in liquid nitrogen (LN2), and stored at −80 °C until RNA isolation. Three independent biological replicates were conducted within each experiment, and each replicate sample consisted of three plants from respective treatments.

### 4.2. Generation of Transgenic Arabidopsis Plants

The full length ORF of *TdGASA1* was cloned into into the *Kpn*I-*Xba*I site of the binary vector pCAMBIA2300, under the control of the 35S promoter (P35S) and then transferred to GV3101 *Agrobacterium* strain. Wild type *Arabidopsis* plants (Col-0) were transformed with the generated binary vector following the floral dip method [53]. Transgenic plants expressing *TdGASA1* were selected on MS/2 medium containing 250 μg/mL of Kanamycin at 22 °C with 70% relative humidity under a long-day photoperiod (16 h of light and 8 h of darkness). The T3 seeds were germinated on MS/2 medium supplemented with 250 µg/mL Kanamycin to confirm transgene stability. Selected seedlings were subsequently transferred to MS/2 medium supplemented with 10 µM ABA, 50 µM MeJA, or 3 mM H_2_O_2_ and treated for 15 days prior to sample collection for further analyses (root length, fresh and dry weight).

Three-day-old seedlings were transferred to MS/2 medium supplemented with 50 µM MeJA and treated for 15 days. Plant materials were harvested and used for RNA extraction and RT-qPCR analysis.

### 4.3. Total RNA Extraction and qPCR Analysis

Total RNA was extracted from durum wheat ‘Karim’ plants subjected to various stress treatments using TRIzol reagent (Invitrogen, Thermo Fisher Scientific, Waltham, MA, USA) according to the manufacturer’s instructions. First-strand cDNA synthesis was performed using 2 μg of total RNA with the enzyme M-MLV reverse transcriptase (Invitrogen, USA). The resulting cDNA was then diluted fivefold prior to qPCR amplification. The amplification was carried out using a Light Cycler 480 (Roche, Basel, Switzerland), with thermal cycling conditions as detailed by Ben Romdhane et al. [54]. To ensure specificity, a melting curve analysis was performed at the end of the qPCR cycle to confirm the presence of a single amplification product. The cycle thresholds (CT) from triplicate PCRs were averaged to quantify transcript levels.

Primers were designed using Primer 3 software (version 4.1.0) to specifically amplify the *TdGASA1* gene and the housekeeping gene cell division control protein (*CDC*, Ta54227), which has been established as the most stable reference gene for normalizing gene expression across different developmental stages and tissues in wheat [55]. The relative expression ratio of the *TdGASA1* gene was calculated using the 2^−ΔΔCt^ method as outlined by Livak and Schmittgen [56]. Each RT-qPCR was performed in triplicate, with three biological replicates for each experimental condition. In addition to the expression analysis of *TdGASA1* transgene in *Arabidopsis*, the expression analysis of several stress-related genes (*AtPDF1.2a*, *AtERF1*, *AtVSP2*, *AtMYC2*, *AtPR1*, *AtACS6*, *AtETR1*, and *AtLOX2*) was conducted in both NT and transgenic *Arabidopsis* plants, following previously established protocols [34]. The gene expression levels were normalized to the tobacco housekeeping *Actin* gene (*NtACT*: AT3G18780) (Table 2).

### 4.4. Pathogenicity Tests

To evaluate the antifungal resistance of transgenic *Arabidopsis* lines, a *Fusarium graminearum* and the *Aspergillus niger* strain (CTM 10099) were cultured as previously described. Spores were harvested and diluted to a concentration of 10^⁵^ spores/mL in a solution containing 0.001% (*v*/*v*) Silwet L-77. A 5 µL aliquot of the spore suspension was then placed at the center of each detached leaf. Control leaves were mock-inoculated with Silwet L-77 solution only. Following inoculation, the leaves were maintained under high humidity at 25 °C, and photographs were taken after five days. The infected leaf area was quantified using ImageJ software version 1.54j (https://imagej.net/ij/, accessed on 16 September 2024). All experiments were performed in triplicate.

### 4.5. Enzymatic Assays

For protein extraction and enzymatic assays, 500 mg of leaves from both control and stressed plants (infected by *Fusarium graminearum* and *Aspergillus niger* for 3 days) were homogenized in 5 mL of extraction buffer (0.1 M potassium phosphate buffer, pH 7.5). The homogenate was then centrifuged at 10,000× *g* for 20 min, and the resulting supernatant was used to measure enzyme activities. CAT activity was determined following the method described by Aebi [57], while SOD activity was measured using the protocol outlined by Wang et al. [58]. POD activity was evaluated using the guaiacol oxidation method, according to Chance and Maehly [59]. The enzyme activity levels in the transgenic lines (TG1 and TG5) were compared to those in non-transgenic (NT) plants through relative quantification.

### 4.6. Cloning and Expression of TdGASA1 Gene in E. coli and Protein Purification

The TdGASA1 protein, originally isolated from durum wheat and identified by Bouteraa et al. [20], was expressed and purified using a recombinant approach. The full-length open reading frame (ORF) of *TdGASA1* was amplified using PfuTurbo DNA polymerase (Stratagene, La Jolla, CA, USA) with primers targeting the 5′ and 3′ ends and incorporating *EcoR*I restriction sites. The *TdGASA1* ORF was then cloned into the *EcoR*I site of the *E. coli* expression vector pET28a, and subsequently transformed into *E. coli* BL21 cells. Then, recombinant proteins were induced with 1 mM IPTG at 30 °C for 6 h and purified using HisLink™ Protein Purification Resin, following the manufacturer’s instructions (Promega). The concentration of the purified proteins was then determined using the Bradford [60] assay, with bovine serum albumin as the standard. The purity and correct size of the recombinant proteins were verified by SDS-PAGE.

### 4.7. In Vitro Antifungal Activity Assay Using Agar Diffusion Method

The potential antifungal activity of TdGASA1 protein was tested on following fungal strains: *Aspergillus niger* (CTM 10099), *Aspergillus flavus* (food isolate), *Apergillus nidulans* (food isolate), *Fusarium graminearum* (ISPAVE 271), *Fusarium oxysporum* (CTM10402), *Fusarium culmorum* (ISPAVE 21w), *Rhizopus nigricans* (CTM10150) and *Alternaria alternata* (CTM 10230). The fungal strains were grown on potato dextrose agar (PDA) (1.5% agar) at 28 °C for 4 days. In each case, inoculum was prepared from an overnight broth culture by its dilution in saline solution to approximately 10^5^ spores/mL.

Antifungal tests were performed by agar well diffusion method as described by Ben Hsouna and Hamdi [61] and broth microdilution assay using potato dextrose agar (Bio-Rad, Marnes-la-Coquette, France). Working cell suspensions were prepared and 100 µL portions were evenly spread onto the surface of the agar plates of potatoes dextrose agar medium (Oxoid Ltd., Basingstoke, UK). Once the plates had been solidified, 06 mm wells were punched into the agar with a sterile Pasteur pipette. The purified *TdGASA1* protein was dissolved in sterile water and 80 μL of solution was poured into the wells. The plates were incubated for 72 h for respective fungal strains at 28 °C. Amphotericin B (10 µg/well) and water served as positive and negative control. Antimicrobial activity was evaluated by measuring the diameter of circular inhibition zones around the well. Tests were performed in triplicate.

### 4.8. Determination of MIC

Minimum inhibitory concentration (MICs) of TdGASA1 was determined according to Ben Hsouna et al. [62] against a panel of microorganisms representing different species of different ecosystems. The test was performed in sterile 96-well microplates with a final volume in each microplate well of 100 µL. A stock solution of the TdGASA1 was prepared in water. The inhibitory activity of the TdGASA1 was properly prepared and transferred to each well in order to obtain a twofold serial dilution of the original sample. To each test well, 10 µL of cell suspension was added to final inoculum concentrations of 10^5^ spores/mL. Positive growth control wells consisted of respective fungal strain only in its optimal medium. Water was used as negative control. The plates were then covered with the sterile plate covers and incubated at 72 h at 28 °C. The MIC was defined as the lowest concentration at which the microorganism does not demonstrate visible growth after incubation. As an indicator of microorganism growth, 25 µL of Thiazolyl Blue Tetrazolium Bromide (MTT), indicator solution dissolved in sterile water were added to the wells and incubated for 30 min [61]. The determination of MIC values was conducted in triplicate.

### 4.9. Data Analysis

Statistical analysis of the data obtained was conducted using SPSS for Windows (V. 12). The data are presented as the mean ± standard error of the mean (s.e.m.) from three independent replicates. To compare the mean values among the groups, two-way analysis of variance (ANOVA) was performed, followed by Duncan test.

## 5. Conclusions

Our comprehensive study revealed that in durum wheat *TdGASA1* gene is an important regulator of reaction to wide range of biotic stress-like stimuli, as well as to a direct infection with fungal pathogens. We therefore analyzed the properties of the purified TdGASA1 protein and found out that it displayed pronounced antimicrobial activity against pathogenic microorganisms. This finding is of considerable importance, as it highlights the potential of TdGASA1 protein as a prospective natural compound for developing antimicrobial agrochemicals or therapeutic agents. Furthermore, the overexpression of *TdGASA1* in transgenic *Arabidopsis* showed that the protective action of *TdGASA1* gene is ubiquitous and attributed to metabolic changes leading to boosted immunity and stress defense machinery. These results may have an important practical impact for developing crops resistant to biotic stresses through genetic engineering.

## Figures and Tables

**Table 1:** Antifungal activity of TdGASA1 and determination of the minimum inhibitory concentrations (MICs) expressed in mg/mL.

Fungal Strain	Inhibition Zones Diameter (mm) ^a^		MIC
	TdGASA1 ^b^	Amphotericin B ^c^	(mg/mL)
*Aspergillus niger* (CTM 10099)	22 ± 0.9	15 ± 0.9	0.625 ± 0.4
*Aspergillus flavus* (food isolate)	20 ± 0.6	10 ± 0.3	0.312 ± 0.6
*Aspergillus nidulans* (food isolate)	18 ± 0.6	0	0.625 ± 0.5
*Aspergillus fumigatus* (food isolate)	18 ± 0.4	0	0.625 ± 0.7
*Fusarium graminearum* (ISPAVE 271)	20 ± 0.4	14 ± 0.5	0.312 ± 0.6
*Fusarium oxysporum* (CTM10402)	17 ± 0.5	14 ± 0.2	0.625 ± 0.5
*Fusarium culmorum* (ISPAVE 21w)	20 ± 0.7	12 ± 0.7	0.312 ± 0.8
*Alternaria alternata* (CTM 10230)	18 ± 0.6	14 ± 0.6	1.25 ± 0.6

Values are given as mean ± SD of triplicate experiments. ^a^ Diameter of inhibition zones including diameter of disc 6 mm. ^b^ TdGASA1: purified protein tested. ^c^ The used concentration of amphotericin B was 10 μg/well.

**Table 2:** Sequences of primers used in PCR and RT-qPCR analysis.

Primers	Sequences
*qTdGASA1-F*	5′-TCCTTTAAAAGAATGCCCAGC-3′
*qTdGASA1-R*	5′-AGTTGTTGTAGCACGGGCAT-3′
*qTdGASA1-EcoRI-F*	5′-TTAGAATTCATGGCTTGTGTCGCACGCA-3′
*qTdGASA1EcoRI-R*	5′-TTACTTAAGTCACGGGCACTTAGGCTTG-3′
*qACT-F*	5′- GTGCCCATTTACGAACGATA-3′
*qACT-R*	5′-GAAGACTCCATGCCGATCAT-3′
*qAtPDF1.2a-F*	5′-CCATCATCACCCTTATCTT-3′
*qAtPDF1.2a-R*	5’-CTGGGAAGACATAGTTGCAT-3’
*qAtLOX2-F*	5’-ATGCTACGTCATGCTGGCTATGGA-3’
*qAtLOX2-R*	5’-TGCCGCTATTATGTATGGCTCCGT-3’
*qAtPR1-F*	5’-ACACCTCACTTTGGCACATC-3’
*qAtPR1-R*	5’-GAGTGTGGAAAACGCAAAGA-3’
*qAtACS6-F*	5′-TTAGCTAATCCCGGCGATGG-3′
*qAtACS6-R*	5′-ACAAGATTCACTCCGGTTCTCCA-3′
*qAtVSP2-F*	5′-CGCCAAATTCTAGTTAAGCACACA-3′
*qAtVSP2-R*	5′-TCGATTGGTGCAACAAATGCT-3′
*qAtERF1-F*	5′-TTCCCTTCAACGAGAACGAC-3′
*qAtERF1-R*	5′-TAGGTTTGTTGCGTGGACTG-3′
*qAtMYC2-F*	5′-AGCAACGTTTACAAGCTTTGATTG-3′
*qAtMYC2-R*	5′-TCATACGACGGTTGCCAGAA-3′
*qAtETR1-F*	5′-CTCCTTCTCCGTCGCTCTC-3′
*qAtETR1-R*	5′-CCTCTCTCACACATACACACAC-3′

## Data Availability

The raw data supporting the conclusions of this article will be made available by the authors on request.

## Supplementary Materials

The following supporting information can be downloaded at: https://www.mdpi.com/article/10.3390/plants14010112/s1, Figure S1: Graphical summary illustrating the experimental workflow.
