# Supplementary figures and images for: Functional Analysis of Durum Wheat GASA1 Protein as a Biotechnological Alternative Against Plant Fungal Pathogens and a Positive Regulator of Biotic Stress Defense
Source: Plants (Basel). 2025 Jan 2;14(1):112. doi: 10.3390/plants14010112 (PMC11723377; doi:10.3390/plants14010112)

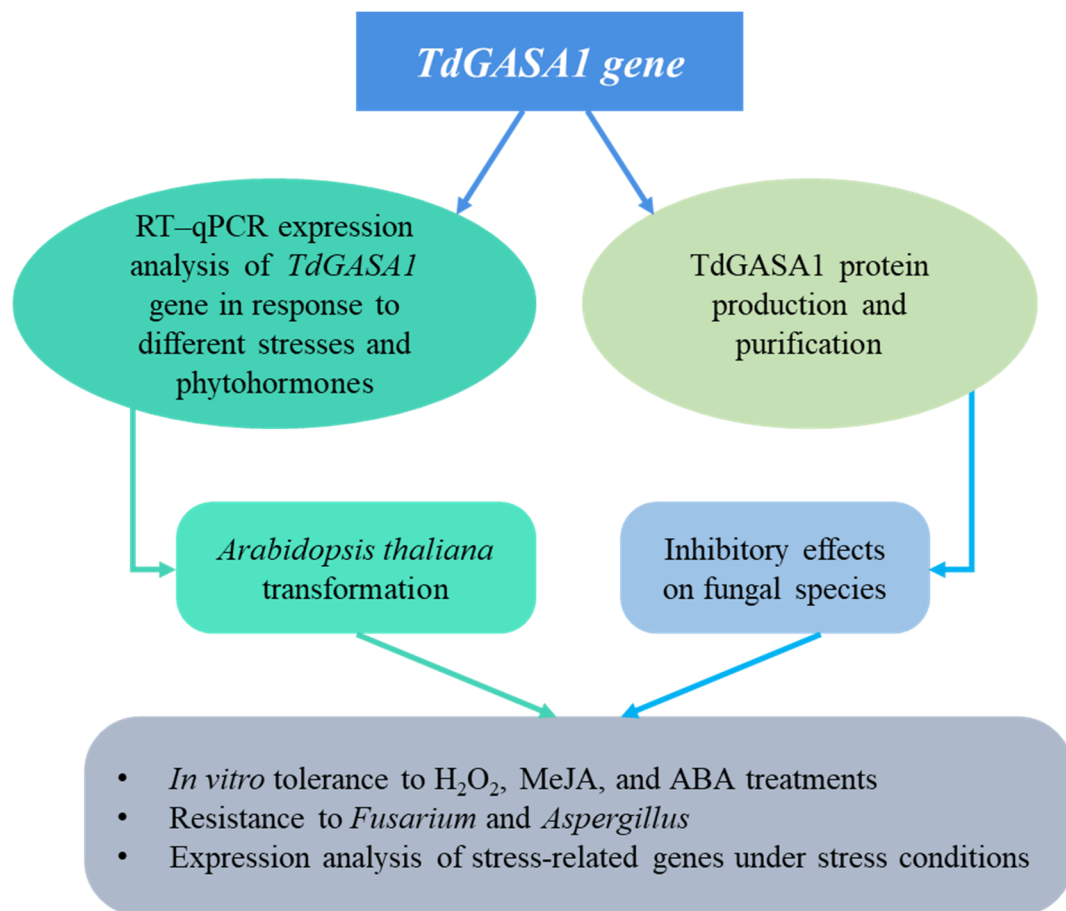

**Figure S1.** Graphical summary illustrating the experimental workflow.

Supplement: Supplementary file 1 [file plants-14-00112-s001.zip › plants-3310865-supplementary.pdf]
